# Pneumodescemetopexy With a Lower Concentration of Perfluoropropane (10% C3F8) in Descemet Membrane Detachment

**DOI:** 10.7759/cureus.16985

**Published:** 2021-08-07

**Authors:** Jun Yong Chow, Ahmad Nurfahmi Akhtar Ali, Mae-Lynn Catherine Bastion

**Affiliations:** 1 Ophthalmology, Universiti Kebangsaan Malaysia Medical Centre, Kuala Lumpur, MYS; 2 Ophthalmology, Hospital Tengku Ampuan Afzan, Kuantan, MYS; 3 Ophthalmology, International Islamic University Malaysia, Kuantan, MYS; 4 Ophthalmology, Hospital Universiti Kebangsaan Malaysia, Kuala Lumpur, MYS

**Keywords:** descemet membrane detachment, pneumodescemetopexy, perfluoropropane, optical coherence tomography, phacoemulsification

## Abstract

A 75-year-old man with underlying left eye idiopathic orbital inflammatory disease and bilateral glaucoma suspect with cup disc ratio 0.7 underwent right eye phacoemulsification. Intraoperatively, Descemet tear and Descemet membrane detachment (DMD) occurred. Pneumodescemetopexy with air bubble was performed. His vision remained counting fingers and the cornea was oedematous three weeks after the operation. Anterior segment optical coherence tomography (ASOCT) showed extensive (80%) rhegmatogenous DMD with planar edge and a maximum of 460 micrometers separation from the stroma. Pneumodescemetopexy with low concentration perfluoropropane (10% C3F8) was performed together with postoperative positioning. On day five post-pneumodescemetopexy, his vision improved to 6/9, the cornea cleared with mild Descemet striae, and the gas bubble reduced to 30% fill. There was no DMD detected on ASOCT. His vision remained 6/9 and the residual gas bubble was 15% in the anterior chamber at two weeks post-pneumodescemetopexy. This case report suggests that pneumodescemetopexy with 10% C3F8 successfully reattached the large nonplanar rhegmatogenous DMD.

## Introduction

Descemet membrane is the basement membrane of corneal endothelium and helps to maintain corneal transparency together with endothelium. It is easily separated from the substantia propria of the cornea. Descemet membrane detachment (DMD) causes corneal edema leading to poor vision or irreversible bullous keratopathy. Sharma et al. reported that almost 43% of cases occurred after cataract surgery [1]. DMD has been classified by Mackool and Holtz into planar (< 1mm separation from stroma) and nonplanar (>1mm separation from stroma) [2], whereas Jacob classification divided DMD into four broad groups, namely rhegmatogenous, tractional, bullous Descemet, and complex Descemet detachment [3]. Various treatment options are available such as observation with topical corticosteroid and hyperosmotic agent, pneumodescemetopexy, viscoelastic or perfluorocarbons liquid tamponade, trans-corneal suture fixation, descemetotomy, interface drainage, manual repositioning, or keratoplasty. Recently, pneumodescemetopexy has become the gold standard treatment for DMD [4]. Pneumodescemetopexy can be performed by using either 100% air, 14-20% sulfur hexafluoride (SF6), or 12-14% perfluoropropane (C3F8). We report a case of nonplanar rhegmatogenous large DMD treated with pneumodescemetopexy by using a lower concentration of 10% C3F8. Until now, there has been no study report regarding pneumodescemetopexy with 10% C3F8 in DMD.

## Case presentation

A 75-year-old gentleman with underlying benign prostate hypertrophy, chronic obstructive pulmonary disease, and hypertension developed a right eye cataract (nuclear sclerosis grade 2) with best-corrected vision acuity (BCVA) of 6/18. His left eye had idiopathic orbital inflammatory disease, and bilateral glaucoma suspect with cup disc ratio of 0.7. The anterior chamber of the right eye was measured to be 2.96mm. He underwent right eye phacoemulsification under topical anesthesia. A clear superior cornea main wound of 2.4mm size and one side port wound were made at ten o’clock and two o’clock respectively. There was a problem with the eyepieces of the operating microscope (Leica M690) intraoperatively causing the surgeon temporarily loss of stereopsis. A Descemet tear was noted at the main wound during insertion of phaco probe. The tear was flattened with ophthalmic viscoelastic device and the surgeon handled the instrument insertion and removal through the wound with extra caution. However, the Descemet membrane detached during main wound stromal hydration. The anterior chamber was then filled with an air bubble of around 80% to reattach the Descemet membrane. Postoperatively, the patient was discharged with topical betagen twice hourly (betamethasone sodium phosphate 0.1%-gentamicin sulphate 0.3%; Duopharma Marketing Sdn. Bhd., Kuala Lumpur, Malaysia) and topical hypertonic saline 5% four times per day.

**Figure 1:**
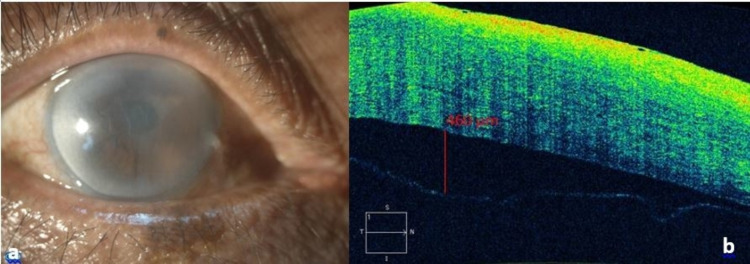
a) Right eye anterior segment picture showed diffuse cornea edematous with Descemet fold except inferotemporal peripheral cornea; b) Anterior segment optical coherence tomography showed Descemet membrane detachment with 460 µm from the stroma. µm: micrometer

At one week post-operation, the patient returned with BCVA of counting fingers close to the face. On examination, multiple microbullae were seen on corneal epithelium, diffuse cornea edema, Descemet folds, and deep anterior chamber with no air bubble seen. DMD was not detected at this time due to hazy cornea. His eye drops were changed to a stronger topical corticosteroid which is dexamethasone 0.1% and continued topical chloramphenicol. The patient was reviewed again three weeks after the operation; his vision remained counting fingers and three-quarters of the cornea involving the visual axis were still severely edematous except inferotemporal cornea. Anterior segment optical coherence tomography (ASOCT) was performed and showed roughly 80% large rhegmatogenous DMD with planar edge and a maximum of 460 micrometers separation from the stroma (Figure 1).

A decision was made to treat with pneumodescemetopexy using a lower concentration perfluoropropane (10% C3F8) under local anesthesia, based on the large nature of the DMD and his status as a glaucoma suspect. Injection of 10% C3F8 gas into the anterior chamber using a 27G needle at eight o’clock via the clear cornea near the limbus was performed under topical anesthesia. This location was chosen as here the Descemet membrane was still well attached to the stroma. C3F8 was allowed to completely fill the anterior chamber for eight minutes, and then one-third of the gas was released to avoid pupillary block glaucoma. Postoperatively, less than 10% of the Descemet membrane remained mildly detached, mainly at the inferonasal part of the cornea. His intraocular pressure (IOP) at one hour and two hours post-operation were normal at 13-15 mmHg. He was advised to maintain a right cheek to pillow position, and to avoid going to locations of high elevation. He was subsequently discharged on the next day with topical corticosteroid and antibiotics as well as topical hypertonic saline.

**Figure 2:**
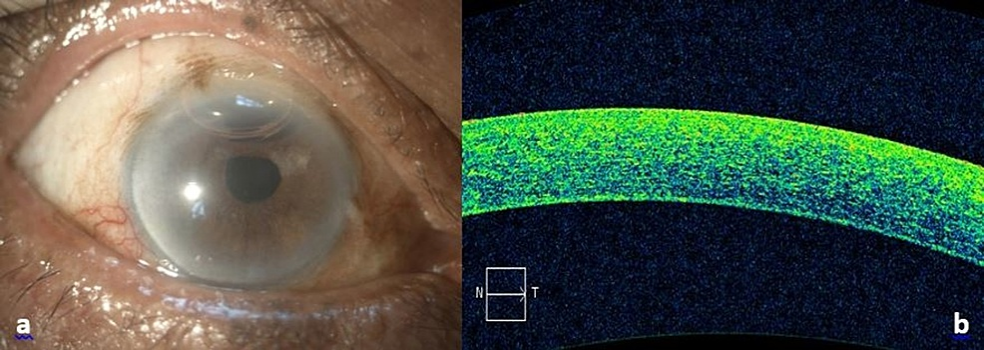
a) Cornea clear with 20% residual gas bubble; b) ASOCT showed Descemet membrane well oppose back to corneal stroma. ASOCT: anterior segment optical coherence tomography

**Figure 3:**
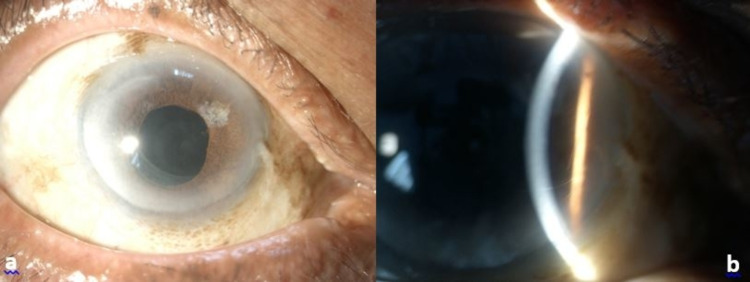
Cornea clear with complete resorption of gas and no re-detached Descemet membrane.

By the fifth day post pneumodescemetopexy, the bubble had contracted in size to just 30% and his cornea was clearer with mild Descemet striae. Hence, his vision had improved to 6/9 as there was no obstruction of the visual axis. The anterior chamber was deep with activity grade 2+, stable posterior chamber intraocular lens, and IOP of 12 mmHg. There was no DMD detected on ASOCT. His vision remained at 6/9 and the residual gas bubble was 20% in the anterior chamber at two weeks post-pneumodescemetopexy (Figure 2). The gas was subsequently completely resorbed. The vision of the patient was 6/6 and the cornea was clear with no DMD at three months follow-up (Figure 3).

## Discussion

Pneumodescemetopexy with C3F8 gas combined with postoperative positioning was selected in this case as this patient had extensive, center-involving, persistent, planar, rhegmatogenous, planar-edged DMD which failed pneumodescemetopexy with air. Furthermore, the only available gas in our hospital was C3F8. However, pneumodescemetopexy with C3F8 is not without complications. Studies have previously reported raised intraocular pressure (13.5%) and pupillary block glaucoma (11%) post pneumodescemetopexy with C3F8 [5,6]. Injection of a large amount of C3F8 can cause compression of the iris against the lens surface leading to iris ischemia and subsequent Urrets-Zavalia syndrome [7]. There was also an animal study that reported endothelial dysfunction after intracameral injection of isoexpansile gases such as C3F8 [8].

Normally, the concentration of C3F8 used for pneumodescemetopexy is 12-14%. However, these concentrations of C3F8 made postoperative raised intraocular pressure a risk for further damage to the optic nerve. Thus, a lower concentration of C3F8 was considered. Several studies found that the nonexpansile concentration of perfluoropropane could be between 10% and 20% [9,10]. In our case, a lower concentration of C3F8 (10%) was used due to the high cup disc ratio of this patient which carried a likelihood of glaucoma. Fortunately, our patient did not have any episode of raised IOP after pneumodescemetopexy. The gas bubble size in the anterior chamber was found to reduce much faster when using a lower concentration of C3F8. This directly hastened the visual recovery as shown in our patient who achieved BCVA of 6/9 with a 30% residual gas bubble in the anterior chamber, at day five post-operation. A lower concentration of C3F8 gas has both advantages of air and gas pneumodescemetopexy. It maintains adequate tamponade in the anterior chamber with a longer duration than air and has less ocular complication than isoexpansile gases. Furthermore, pneumodescemetopexy with 10% C3F8 was found to successfully reattach a chronic DMD. The DMD was initially missed one week after the operation but it did not have severe consequences on the reattachment of the DM. In fact, our group (Bastion et al.) describes a late reattachment after 10 weeks from the phacoemulsification [11]. However in such late cases, then it may be necessary to perform a venting incision. In this case, we managed to treat the patient who had DMD for three weeks successfully without a corneal venting incision. Besides, a 10% concentration of C3F8 is easier to prepare than 12% or 14%. Therefore, 10% C3F8 can be considered in patients who are susceptible to glaucoma or DMD cases that failed air pneumodescemetopexy.

## Conclusions

Our case report reveals that pneumodescemetopexy with 10% C3F8 is successful to reattach extensive, persistent, nonplanar rhegmatogenous DMD. A lower concentration of C3F8 can be considered in the cases when patients are susceptible to glaucoma, patients that have logistic issues for repeat air pneumodescemetopexy, and precious eyes that require early visual recovery. It has an adequate tamponade effect yet fewer ocular complications such as raised IOP and vision disturbances.
